# Bone Regeneration After Maxillary Sinus Augmentation with Allogeneic and Xenogeneic Biomaterials with Adjunctive Photobiomodulation: Histological and Radiological Secondary Outcomes of a Randomized Clinical Trial

**DOI:** 10.3390/jfb17040186

**Published:** 2026-04-10

**Authors:** Sebastian Dominiak, Aleksandra Piotrowska, Marzena Dominiak, Tomasz Gedrange, Piotr Dzięgiel, Alicja Baranowska, Michał Ciszyński, Jakub Hadzik, Paweł Kubasiewicz-Ross

**Affiliations:** 1 Department of Dental Surgery, Wroclaw Medical University, Krakowska 26, 50-425 Wroclaw, Polandpawel.kubasiewicz-ross@umw.edu.pl (P.K.-R.); 2 Division of Histology and Embryology, Department of Human Morphology and Embryology, Wroclaw Medical University, 50-368 Wroclaw, Poland

**Keywords:** maxillary sinus augmentation, bone regeneration, bone substitutes, low-level light therapy, xenografts, allografts, dental implants

## Abstract

Background: Atrophy of the alveolar ridge in the posterior maxilla often requires sinus floor elevation prior to implant placement. Photobiomodulation using low-level laser therapy (LLLT) has been suggested as a supportive approach for bone healing, although data based on histological evaluation are still limited. Methods: This study presents histological and radiological secondary outcomes of a randomized clinical trial on bone regeneration after lateral window sinus augmentation. Twenty patients were allocated according to grafting material (allogeneic or xenogeneic) and the use of adjunctive LLLT. After 6 months, bone core biopsies were obtained at the time of implant placement and processed for histological analysis. Radiological bone gain was assessed using CBCT. Results: Bone gain was achieved in all groups, allowing implant placement in every case. Mean bone gain reached 7.53 ± 3.32 mm in LLLT-treated sites and 7.02 ± 2.00 mm in controls, with no statistically significant differences. Histological analysis confirmed trabecular bone formation across all groups. Mild inflammatory cell infiltrates were observed more frequently in LLLT-treated sites (*p* = 0.029), although this finding was not associated with impaired tissue organization or compromised healing. Conclusions: Both allogeneic and xenogeneic grafts showed good biocompatibility and supported effective bone regeneration after sinus augmentation. The addition of photobiomodulation did not demonstrate statistically significant clinical or radiological benefits within this exploratory cohort, but it may be associated with subtle differences in tissue remodeling.

## 1. Introduction

Reconstruction of insufficient alveolar bone height in the posterior maxilla remains a common challenge in implant dentistry. Post-extraction bone loss, combined with sinus pneumatization, reduces the available vertical dimension and may prevent predictable implant placement. In such cases, maxillary sinus augmentation remains the treatment of choice [1].

The procedure was originally described by Tatum and later modified by Boyne and James [1,2]. In cases of advanced bone atrophy, a lateral window approach is typically recommended. This technique involves elevation of the Schneiderian membrane, creating a well-vascularized compartment that provides favorable osteoconductive conditions for the placement of graft material [1]. However, the choice of surgical approach remains influenced by residual bone height and anatomical variability, and considerable heterogeneity in clinical decision-making persists [3].

Various materials, including freeze-dried bone allograft, β-calcium phosphate tri-basic and xenografts, such as deproteinized bovine bone mineral, have been proposed as bone substitutes that can be applied during the sinus augmentation procedure. The choice depends on biological properties, clinician experience and ethical considerations. In recent years, in practice, allografts and xenografts have been established as a bio-material of choice for that purpose. Allografts are often preferred due to their favorable biocompatibility and predictable remodeling pattern. Moreover, their structural similarity to human bone supports osteoconduction and to some degree osteoinduction [4,5]. On the other hand, xenografts are known for their high volumetric stability and very slow resorption rate, which helps maintain the three-dimensional scaffold necessary for prolonged osteoconduction [6]. Despite the generally favorable long-term outcomes, the sinus-lift procedure carries a risk of postoperative complications. Moreover, it requires a prolonged healing period. These factors elevate biological costs and might diminish patients’ overall satisfaction. Therefore, efforts have been made to identify adjunctive methods that could support the postoperative phase, reduce the incidence of complications and shorten the healing time.

One of the possible options for that purpose is low-level laser therapy (LLLT), which is a form of photobiomodulation. It acts on cells through mitochondrial light absorption. This process enhances ATP production, modulates cytokine release, and stimulates fibroblast and osteoblast proliferation [7,8]. Previous reports have demonstrated that LLLT enhances cellular metabolism, angiogenesis, and early osteoblastic activity, which may contribute to improved vascularization and biological integration of grafted bone in regenerative procedures [9]. Despite its growing popularity, evidence specifically addressing the use of LLLT in maxillary sinus augmentation procedures remains limited. To date, only a few studies have investigated its potential effects in sinus augmentation post-op period, suggesting improvement in radiographic bone healing [10,11].

## 2. Materials and Methods

### 2.1. Study Design

This study reports secondary outcomes from the randomized clinical trial entitled “Assessment of Anti-Human Leukocyte Antigen (Anti-HLA) Antibody Development Following Bone Regeneration Procedures Prior to Dental Implant Therapy.” The clinical trial was conducted at the Medical Innovation Center Wrocław (MCIW), which serves as the clinical base of the Department of Oral Surgery, Wrocław Medical University (Wrocław, Poland), between 2023 and 2024. The histological and radiological outcomes analyzed in the present study were predefined as secondary endpoints in the original study protocol.

The study protocol was approved by the Bioethics Committee of Wrocław Medical University (Approval No. KB 10/2023N). All participants provided written informed consent for both the surgical procedure and participation in the study. The investigation was conducted in accordance with the Declaration of Helsinki and applicable data protection regulations (GDPR).

**Figure 1:**
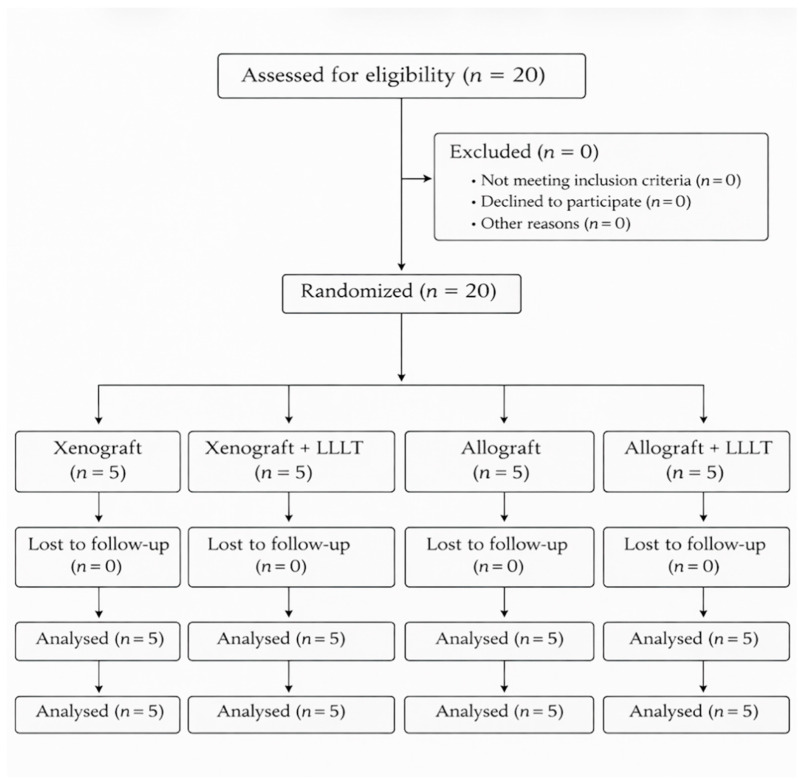
CONSORT flow diagram showing patient enrollment, randomization, allocation to study groups, follow-up, and analysis.

The trial was registered at ClinicalTrials.gov (Identifier: NCT07474857). The study design and reporting followed the CONSORT guidelines for randomized clinical trials, and the patient enrollment process is presented in the CONSORT flow diagram (Figure 1).

The study was supported by a doctoral research subsidy from Wrocław Medical University (grant no. SUBK.B040.23.051).

### 2.2. Study Population and Eligibility Criteria

Participants were consecutively recruited among patients seeking implant-supported rehabilitation in the posterior maxilla at the MCIW Dental Clinic, which serves as the clinical base of the Department of Oral Surgery, Wrocław Medical University (Wrocław, Poland), between 2023 and 2024. The study was conducted as a single-center clinical investigation. All patients required bone augmentation due to insufficient bone height in the posterior maxilla prior to dental implant placement.

A total of 20 patients requiring maxillary sinus augmentation using the lateral window technique as part of their implant treatment plan were enrolled in the study. Patient recruitment and eligibility assessment were performed by an investigator who was not involved in performing the surgical procedures (A.B.).

The inclusion criteria were as follows: (1) age ≥ 18 years and the ability to provide informed consent for the surgical procedure; (2) partial edentulism in the posterior maxilla with one or more missing teeth; (3) residual alveolar ridge height < 5 mm and ridge width > 7 mm in the region planned for maxillary sinus augmentation; and (4) at least 2 mm of keratinized tissue height (HKT) in the region of interest to suport wound primary closure.

The exclusion criteria included poor oral hygiene (plaque index > 20%), pregnancy or breastfeeding, previous sinus surgery in the treated area, active sinus infection or large sinus cysts, and general or local contraindications to oral surgical procedures, including uncontrolled systemic diseases affecting bone metabolism, immunocompromised states, severe cardiovascular conditions, or previous radiation therapy in the head and neck region.

All eligible patients received detailed information about the study and provided written informed consent prior to enrollment.

### 2.3. Randomization and Allocation

Participants were randomly allocated to one of four parallel study groups using a simple randomization procedure performed by an independent investigator not involved in the surgical procedures (A.B.). Randomization was carried out after confirmation of eligibility criteria and obtaining informed consent. Group assignments were placed in sealed opaque envelopes, which were opened after patient enrollment, ensuring allocation concealment.

The allocation followed a 1:1:1:1 ratio, resulting in four groups defined by the type of bone grafting material and the application of adjunctive low-level laser therapy (LLLT). The study followed a 2 × 2 factorial design, with two factors: the type of bone grafting material (xenogeneic vs. allogeneic) and the use of adjunctive photobiomodulation (LLLT).

### 2.4. Study Groups and Intervention

Participants were randomly allocated to four study groups (*n* = 5 each) according to biomaterial type (xenogeneic or allogeneic) and the use of adjunctive low-level laser therapy (LLLT).

One group received an allogeneic cortico-cancellous bone graft in the form of granules with a granulometry of 0.5 mm (Biobank, Lieusaint, France), while another group received a xenogeneic spongious bone graft in the form of granules with a granulometry of 0.25–1.0 mm (Geistlich, Wolhusen, Switzerland).

Accordingly, four study groups were established, each consisting of five patients:

G1—Xenograft,
G2—Xenograft + LLLT,
G3—Allograft,
G4—Allograft + LLLT.

### 2.5. Preoperative Assessment

All patients underwent a comprehensive clinical examination to determine indications and contraindications for the sinus floor elevation procedure. Subsequently, radiological assessment was performed using cone-beam computed tomography (CBCT) acquired with the Planmeca Viso^®^ G7 system (Planmeca^®^, Helsinki, Finland).

The field of view (FOV) was centered on the maxilla and measured 300 mm in width and 200 mm in height. CBCT scans were analyzed using Romexis software release 6.0 (Planmeca^®^, Helsinki, Finland). The following parameters were evaluated: residual bone height (RBH), the condition of the maxillary sinus, including Schneiderian membrane thickening; the presence of sinus polyps, cysts or septa; and possible periapical inflammatory lesions of adjacent teeth.

All measurements were performed by an investigator who was not involved in the surgical procedures (A.B.).

### 2.6. Surgical Procedure

All surgical procedures were performed under local anesthesia using 4% articaine with epinephrine 1:200,000 (Septanest^®^ 1:200,000, Septodont, Saint-Maur-des-Fossés, France). A full-thickness mucoperiosteal flap with a distal releasing incision was elevated to expose the lateral wall of the maxillary sinus.

A lateral antrostomy was performed using the DASK^®^ sinus kit (Dentium^®^, Suwon, Republic of Korea). An osteotomy window of standardized dimensions was created with an 8 mm diamond drill under constant saline irrigation and minimal pressure. The Schneiderian membrane was then carefully elevated using dedicated sinus elevators to create a recipient site for the graft material.

**Figure 2:**
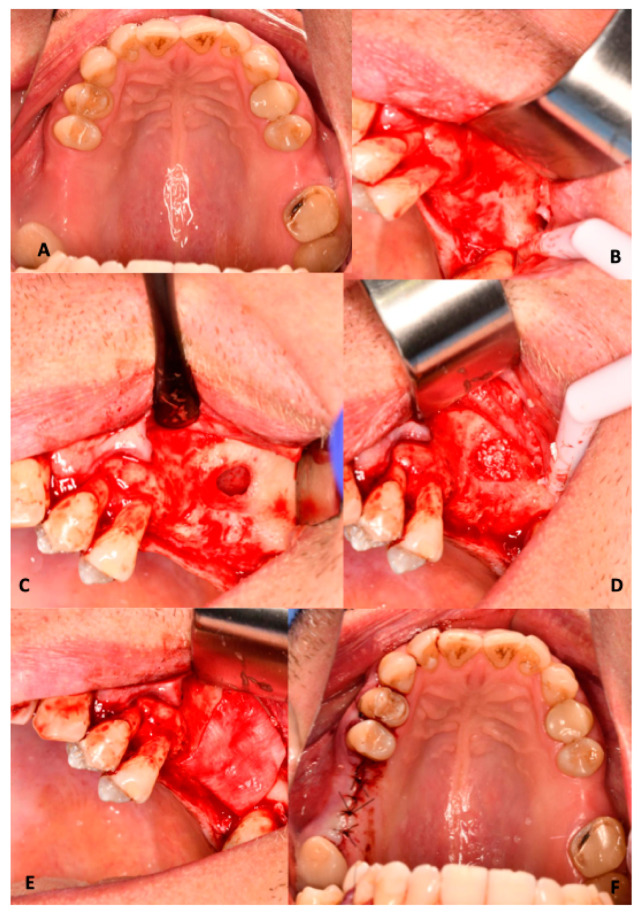
Lateral window maxillary sinus augmentation procedure. (**A**) Preoperative clinical view of the surgical site. (**B**) Elevation of a full-thickness mucoperiosteal flap. (**C**) Preparation of the lateral antrostomy to access the maxillary sinus. (**D**) Placement of the graft material within the elevated sinus cavity. (**E**) Coverage of the antrostomy with a resorbable collagen membrane. (**F**) Flap repositioning and tension-free suturing.

Depending on group allocation, the sinus cavity was filled with the assigned bone graft material. The augmented area was covered with a resorbable collagen membrane (Bio-Gide^®^, Geistlich, Wolhusen, Switzerland) to prevent soft tissue ingrowth. The amount of graft material was determined intraoperatively based on the size of the created sinus cavity and the extent of membrane elevation. The volume of graft material was not quantitatively recorded. The flap was repositioned and sutured without tension using non-resorbable 5/0 nylon monofilament sutures (Atramat^®^, Mexico City, Mexico) (Figure 2).

### 2.7. Photobiomodulation

In the designated groups (+LLLT), adjunctive low-level laser therapy was applied using a diode laser device (Lasotronix^®^, Piaseczno, Poland) with a wavelength of 635 nm and a beam diameter of 0.5 cm^2^. Laser irradiation was performed intraorally with the tip positioned over the osteotomy site and the overlying soft tissues.

The irradiation parameters were 6 J/cm^2^ delivered for 1 min in continuous mode. The procedure was performed immediately after surgery and repeated on postoperative days 3 and 7. The selected irradiation parameters were standard protocols and fall within the commonly reported therapeutic range for intraoral applications (630–660 nm, 4–10 J/cm^2^) [12].

### 2.8. Postoperative Management and Follow-Up

All patients received identical postoperative care regardless of the study group. Patients were prescribed an antibiotic regimen (Duomox^®^, Astellas Pharma, Europe B.V., Leiden, The Netherlands) at a dose of 2.0 g per day and were instructed to use chlorhexidine mouthwash (Eludril Classic®, Pierre Fabre, Castres, France) twice daily for two weeks.

Sutures were removed seven days after surgery. Follow-up visits were scheduled on postoperative days 3 and 7 to assess wound healing and possible complications.

A radiological follow-up examination was performed 6 months after surgery using CBCT imaging to evaluate bone regeneration.

### 2.9. Implant Placement and Biopsy Collection

Six months after the maxillary sinus augmentation elevation procedure, patients returned for implant placement surgery. Under local anesthesia (4% articaine with epinephrine 1:200,000), a full-thickness mucoperiosteal flap without vertical releasing incisions was elevated.

A trephine bur from the Khoury–Meisinger trephine kit (Hager & Meisinger GmbH, Neuss, Germany) was used to harvest a cylindrical bone biopsy containing both newly formed bone and graft material. The specimen was immediately fixed in 4% formalin and transported to the Department of Histology and Embryology, Wrocław Medical University.

Implant osteotomy preparation was completed using the MIS C1 XD drilling system (MIS, Misgav, Israel). Implant length was selected individually based on the available bone height after augmentation, following standard clinical protocols, with preference for implants in cases with limited vertical bone availability. When required, minor transcrestal sinus floor elevation was additionally performed to achieve adequate primary stability, particularly in borderline situations with reduced residual bone height.

**Figure 3:**
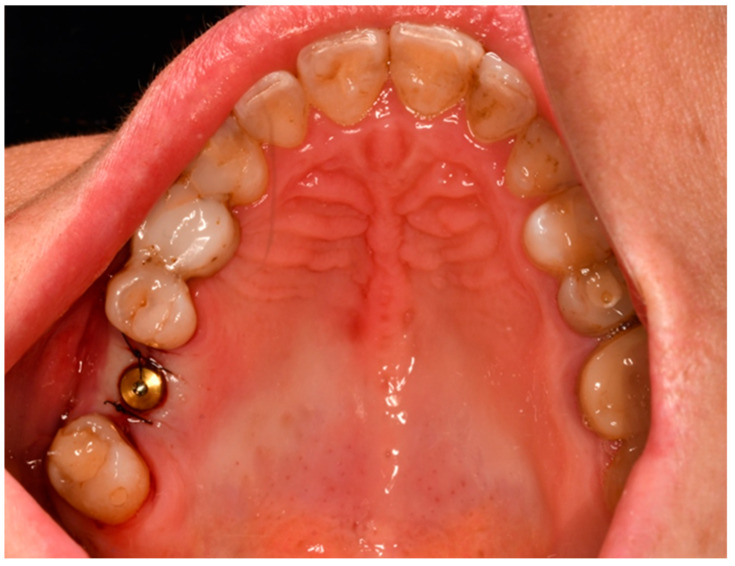
Clinical view of an MIS implant (MIS Implants Technologies Ltd., Bar Lev Industrial Park, Israel) with CONNECT abutment and healing cap immediately after placement.

Dental implants were inserted manually using the manufacturer’s insertion tools and torque ratchet to ensure controlled positioning. A CONNECT abutment with a healing cap was placed, and the flap was sutured around the implant for open healing using non-resorbable 5/0 nylon sutures (Atramat^®^, Mexico City, Mexico) (Figure 3).

### 2.10. Histological Evaluation

Histological evaluation was performed independently by two authors not involved in the clinical part of the study (A.P. and P.D.). The assessors were blinded to group allocation. In cases of discrepant scores, the final score was established by consensus. The analysis was conducted in the Division of Histology and Embryology of Wroclaw Medical University using the Olympus BX41 microscope (Olympus, Tokyo, Japan) at 20× magnification.

The material, fixed in a 4% buffered formalin solution, was rehydrated and decalcified in 10% ethylenediaminetetraacetic acid (EDTA) and then embedded in paraffin blocks. Histological slides, 4 µm thick, were prepared from these blocks and stained using the hematoxylin-eosin staining.

**Figure 4:**
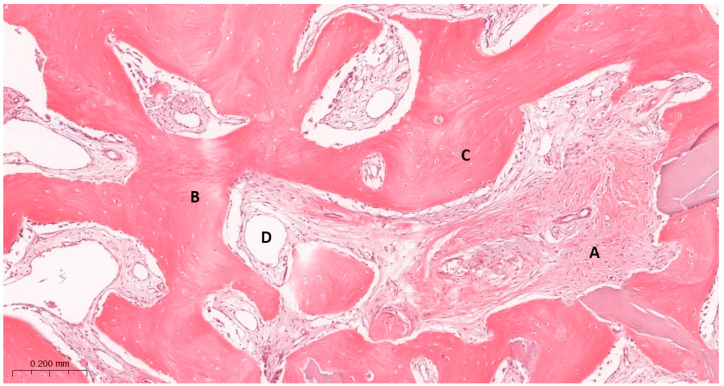
Reference histological image illustrating BM and TB/CT scoring criteria. Micrograph of new alveolar bone. Primary bone (B) is rich in osteocytes (C) and is accompanied by connective tissue (A) with numerous blood vessels (D). H&E staining. Magnification ×100. This image is representative of a BM score of 1 and TB/CT score of 1, indicating predominance of well-organized trabecular bone with minimal connective tissue.

During the histological evaluation, the following parameters were assessed:

• Inflammatory cell infiltrates (ICIs) were quantified as the number of inflammatory cell infiltrates per high-power field (HPF). The following scoring was applied: 0 points for fewer than one ICI per HPF, 1 point for fewer than three ICI per HPF, 2 points for fewer than five ICI per HPF, and 3 points for fewer than eight ICI per HPF.
• The degree of tissue mineralization and structural maturation was evaluated using the Trabecular Bone-to-Connective Tissue Ratio (TB/CT). This parameter described the relative proportion of trabecular bone to loose connective tissue and was scored as follows: 1 point when trabecular bone predominated over loose connective tissue, 2 points when both components were present in comparable amounts, and 3 points when loose connective tissue predominated over trabecular bone.
• Bone morphology (BM) was assessed to characterize the structural pattern of the regenerated bone. The scoring reflected the presence and distribution of mineralized tissue: 1 point was assigned when numerous large bone areas with few small bone islands were observed, 2 points when moderately numerous large bone areas and small bone islands were present, and 3 points when large bone areas were scarce but numerous small bone islands were identified (Figure 4).

Inflammatory cell infiltrates were included as an indicator of local tissue response to the graft material and the healing process, reflecting inflammatory activity and tissue remodeling during bone regeneration.

### 2.11. Radiological Evaluation

Radiological assessment was performed using CBCT imaging and Romexis software (version 6.0; Planmeca Oy, Helsinki, Finland). Bone gain (BG) was measured at the planned implant site in standardized cross-sectional views along a vertical axis perpendicular to the alveolar crest. Residual bone height (RBH) and postoperative bone height were assessed at the same location, and BG was calculated as their difference. Radiological assessment was performed by an investigator not involved in the surgical procedures, ensuring blinding.

### 2.12. Statistical Analysis

Statistical analysis was performed using STATISTICA software (version 13.3, TIBCO Software Inc., Palo Alto, CA, USA). Due to the small sample size and non-normal distribution of the variables, non-parametric tests were applied, including the Mann–Whitney U test for group comparisons. Categorical variables were analyzed using appropriate tests of independence. Statistical significance was set at *p* < 0.05.

The study was designed as an exploratory 2 × 2 factorial investigation. Due to the small subgroup size (*n* = 5), comparisons were performed separately for the main factors (graft type and LLLT), and no interaction or four-group analyses were conducted. No formal sample size calculation was performed due to ethical constraints related to bone biopsy collection.

## 3. Results

All participants tolerated the procedures well, and no major complications were observed in any of the study groups. All patients attended the scheduled follow-up visits and completed the study protocol, including dental implant placement.

### 3.1. Demographic Characteristics

In groups G1 and G3, the majority of participants were male, whereas in groups G2 and G4, women predominated.

The mean age varied between groups, ranging from approximately 45 years in G1 and G2 to about 52 years in G3 and slightly over 59 years in G4. However, these differences were not statistically significant (Table 1).

### 3.2. Radiological Evaluation

Substantial bone gain (BG) was observed in all groups, allowing for subsequent implant placement. The results of the radiological evaluation are presented in Table 1, Table 2 and Table 3.

#### 3.2.1. Residual Bone Height

**Figure 5:**
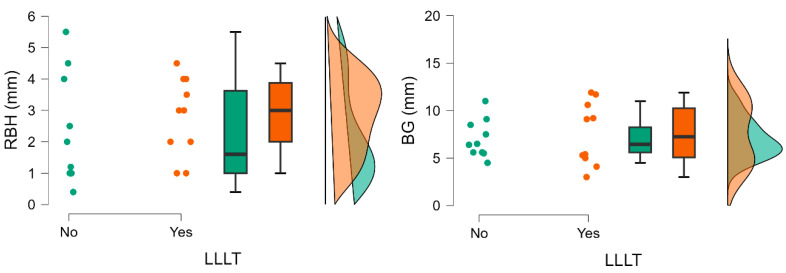
Comparison of radiological parameters in groups of patients after sinus-lift surgical procedures differing in low-level laser therapy.

**Figure 6:**
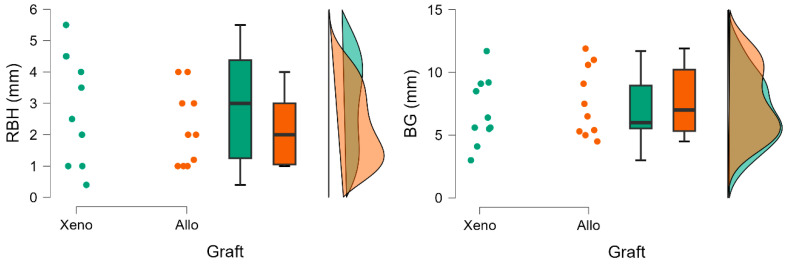
Comparison of radiological parameters in groups of patients after sinus-lift surgical procedures differing in graft material.

Baseline residual bone height (RBH) values were comparable between groups regarding LLLT adjunctive therapy (2.80 ± 1.25 vs. 2.31 ± 1.76) and bone graft (2.89 ± 1.76 vs. 2.00 ± 1.21), demonstrating baseline anatomical similarity. No statistically significant differences between groups were observed (Table 2 and Table 3) (Figure 5 and Figure 6).

#### 3.2.2. Bone Gain

Bone gain (BG) was observed in all groups, regardless of the grafting material used or the application of adjunctive LLLT. The LLLT groups expressed higher levels of BG (7.53 ± 3.32 vs. 7.02 ± 2.00), however, with greater variability. Despite higher levels of RBH in xenograft groups, BG was higher in groups where an allograft was used (7.68 ± 2.77 vs. 6.87 ± 2.67). However, these differences were not statistically significant (Table 2 and Table 3; Figure 5 and Figure 6).

### 3.3. Results of the Histological Study

**Figure 7:**
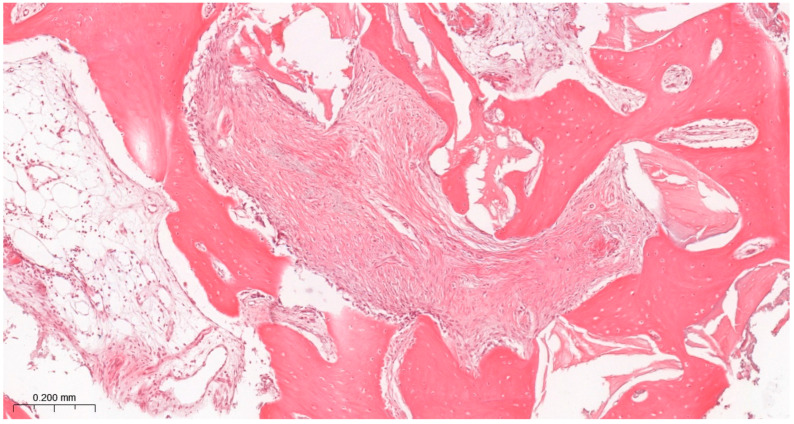
Results of histological assessment of a G1 participant. A high amount of connective tissue is accompanied by poorly organized bone trabeculae. H&E staining. Magnification ×100. This image is representative of a pattern corresponding to BM scores of 2 and 3 and TB/CT scores of 2 and 3, reflecting less organized bone structure with a higher proportion of connective tissue.

**Figure 8:**
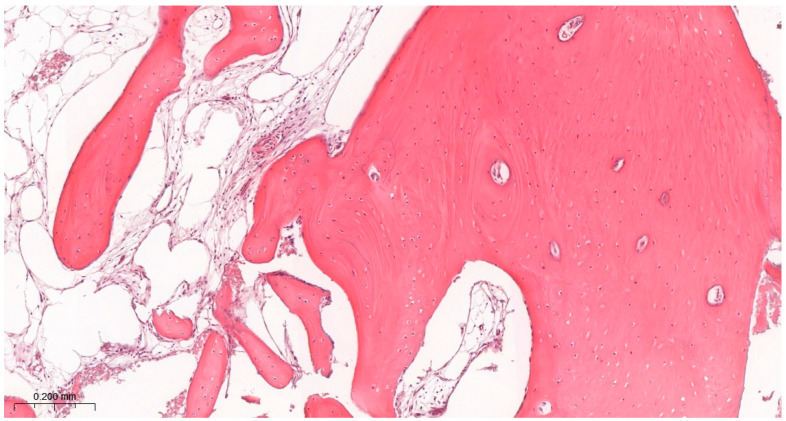
Results of histological assessment of a G4 participant. Well-organized trabecular bone accompanied by highly vascularized connective tissue. H&E staining. Magnification ×100. This image is representative of a BM score of 1 and TB/CT score of 1, indicating well-organized trabecular bone and advanced tissue maturation.

Bone tissue was observed in all samples (Table 1), indicating successful bone regeneration following sinus floor augmentation with both grafting materials (Figure 7 and Figure 8).

The detailed results of the histological evaluation are presented in Table 3.

#### 3.3.1. Inflammatory Cell Infiltrates

All groups exhibited low inflammatory activity. Scores ranged predominantly between 0 and 1, with no sample exceeding two points. Mild inflammatory infiltrates were more frequent in the photobiomodulation-treated groups (60% vs. 10%). However, a statistically significant difference was detected (*p* = 0.029); despite this, inflammatory cell infiltration remained low across all groups, suggesting limited clinical relevance of this finding (Table 4).

#### 3.3.2. Trabecular Bone-to-Connective Tissue Ratio

Most samples were scored with comparable amounts of trabecular bone and loose connective tissue, corresponding to intermediate levels of regeneration. Statistical analysis showed no significant differences among groups (Table 4) (Figure 7 and Figure 8).

#### 3.3.3. Bone Morphology

Bone morphology was characterized by a combination of larger mineralized regions and smaller trabecular structures typical of intermediate-stage regeneration at 6 months. Scores remained consistent across groups. Moreover, no significant differences were identified. However, qualitative assessment suggested that LLLT groups showed more uniform and structurally consistent trabeculation (80% vs. 70%) (Table 4) (Figure 7 and Figure 8).

## 4. Discussion

The role of photobiomodulation in sinus-lift procedures remains incompletely defined, particularly in studies that include histological evaluation. Direct comparison with previous reports is difficult due to substantial methodological heterogeneity, including differences in grafting materials, surgical techniques, stabilization protocols, observation periods, and outcome measures [4,6,13]. Moreover, only a limited number of studies have specifically examined low-level laser therapy (LLLT) as an adjunct in sinus augmentation in conjunction with histological analysis.

The primary goal of bone substitute use in maxillary sinus floor elevation is to achieve sufficient bone quality and volume to allow predictable implant placement. This is essential because only properly mineralized bone allows for direct and functional bone-to-implant contact [4,5,6,14]. In regenerative bone procedures, there is always a concern that, instead of forming mature and well-organized bone, healing may result in a heterogeneous fibro-osseous tissue. The quality of newly formed bone remains a key determinant of clinical success in alveolar bone augmentation procedures, as highlighted in recent studies [15]. Although such structures may radiographically mimic successful outcomes through increased ridge height or radiodensity, they do not necessarily provide an adequate structural foundation for implant placement. Therefore, histological evaluation remains a critical tool for assessing the true quality of regenerated tissue [16,17].

In our study, generally favorable outcomes were achieved for both types of bone substitute materials. Histological and radiological findings indicated that the newly formed tissue was suitable for subsequent implant placement, and only minor differences were observed between groups. These findings are consistent with previous reports on sinus augmentation performed without adjunctive therapy. Mazor et al. reported residual bone height of approximately 2.9 (±0.9) mm and bone gain of 10.1 (±0.9 mm), whereas Tajima et al. observed an increase in bone height from 4.28 ± 1.00 mm to 11.8 ± 1.67 mm following augmentation [18,19]. Similarly, earlier studies have demonstrated comparable performance of allogeneic and xenogeneic graft materials in terms of new bone formation and histomorphometric outcomes [14,20].

Despite the generally comparable outcomes between groups, one statistically significant difference was observed in the histological analysis. The only statistically significant histological difference concerned inflammatory cell infiltrates, which were more frequently observed in the LLLT-treated sites. Importantly, these infiltrates remained mild and were not associated with histological evidence of poor tissue integration, excessive fibrous healing, or radiologically inferior augmentation outcomes. In this context, the finding should not be interpreted as evidence of a harmful pro-inflammatory effect of photobiomodulation, but rather as a possible indicator of altered healing kinetics or prolonged remodeling activity within the augmented sinus. Considering that biopsies were obtained 6 months after surgery, the presence of sparse inflammatory cells may reflect ongoing biological activity during tissue maturation rather than clinically relevant inflammation. Nevertheless, this observation warrants further investigation in studies with larger cohorts and more detailed immunohistochemical characterization of the inflammatory infiltrate.

The lack of statistically significant differences between groups may be explained by the inherently favorable biological environment of the maxillary sinus, characterized by high vascularization and osteoconductive conditions. Such an environment may limit the detectable additive effect of photobiomodulation, as the baseline regenerative potential is already high.

Bone gain values in the LLLT-treated groups were slightly higher than in controls, although the differences were not statistically significant. This trend may suggest a potential influence of photobiomodulation on early phases of bone healing. Previous studies have reported that LLLT can enhance cellular metabolism, angiogenesis, and osteoblastic activity, which may contribute to improved integration of grafted material [21,22,23]. Similar CBCT-based approaches have been used to evaluate dimensional bone changes following alveolar augmentation procedures [24]. In a similar context, experimental studies on titanium implant surfaces have shown that physicochemical modifications, including anodization combined with plasma-based treatments, can improve cellular adhesion and biological responses at the implant–tissue interface [25].

In one of the few histomorphometric studies evaluating LLLT as an adjunct in sinus-lift procedures, conducted on eight participants using a split-mouth design with FDBA and PRF, irradiated sites demonstrated significantly greater new bone formation and higher radiological density compared with controls [23]. A more recent study suggested that photobiomodulation at 660 nm and 808 nm may promote higher bone tissue content and more advanced maturation of regenerated tissue in xenograft-based sinus augmentation, although no differences in immunoreactive cell concentrations were observed [26]. However, this study did not include radiological evaluation and was limited by a very small sample size. In contrast, another study using LLLT at 620 ± 2 nm reported no significant differences in inflammation, bone quality, or collagen maturity following sinus-lift procedures [27].

In this context, the wavelength used in this study (635 nm) falls within the red-light range commonly applied in intraoral low-level laser therapy (LLLT) protocols and supported by previous evidence [28]. However, near-infrared wavelengths are known to provide greater tissue penetration. In the context of sinus augmentation, this may be clinically relevant, as the target area extends into the deeper grafted compartment. Therefore, the lack of a measurable benefit observed in the present study may be related to limited penetration depth rather than the absence of a biological effect. Moreover, current evidence highlights considerable heterogeneity in LLLT parameters, including wavelength, which may influence treatment outcomes [12].

Several limitations of the present study should be acknowledged. The relatively small sample size reduces statistical power and limits the ability to detect subtle differences that may still be clinically relevant, which is why the present study should be interpreted as hypothesis-generating rather than confirmatory. However, similar constraints are commonly encountered in studies involving bone biopsy collection after sinus augmentation due to ethical and logistical considerations [14,20]. Current methodological recommendations indicate that larger sample sizes are required to achieve adequate statistical power in dental research [29]. In addition, despite randomization, individual variability in sinus anatomy and healing capacity may have influenced the observed outcomes.

The six-month observation period represents another limitation. Although commonly used in studies evaluating early bone regeneration, sinus augmentation is a dynamic and long-term process. Longer follow-up, including implant stability measurements and extended radiological or histomorphometric assessment, would provide a more comprehensive evaluation of bone maturation. Furthermore, the lack of immunohistochemical analysis limits the ability to characterize the biological nature of the observed inflammatory infiltrates. Long-term implant success is also influenced by additional factors, including loading protocols and peri-implant bone stability, as reported in studies evaluating implants placed in augmented maxillae [30,31]. In addition, the volume of graft material was not quantitatively recorded, as it was determined intraoperatively based on the size of the sinus cavity, which may have contributed to variability in bone gain outcomes. Moreover, the absence of histomorphometric and micro-computed tomography (micro-CT) analyses represents a limitation, as these methods could provide a more detailed quantitative and three-dimensional assessment of bone regeneration [32].

Overall, the findings indicate that photobiomodulation did not demonstrate a measurable clinical or radiological advantage within the limitations of this exploratory study. At the same time, its influence on the biological aspects of tissue remodeling cannot be excluded, as suggested by the histological observations.

## 5. Conclusions

Both xenogeneic and allogeneic graft materials, with or without adjunctive LLLT, proved effective and safe for maxillary sinus augmentation. Although photobiomodulation did not translate into statistically significant differences, consistent trends across radiological and histological parameters suggest a potential modulatory effect on bone healing dynamics.

## Figures and Tables

**Table 1:** Overall summary of the study results.

Group	Age	Gender	ICI	TB/CT	BM	RBH	BG
1	47	M	1	1	2	0.4	6.4
1	59	F	0	1	2	2.5	5.6
1	38	M	0	1	1	1	5.5
1	47	M	0	2	1	4.5	8.5
1	33	F	0	1	1	5.5	5.6
2	33	F	0	2	1	1	4.1
2	50	F	0	1	1	2	9.1
2	52	F	1	1	2	4	3
2	42	F	1	3	1	4.5	11.7
2	44	M	1	1	1	3.5	9.2
3	46	F	0	2	2	2	9.1
3	59	M	0	1	1	4	6.5
3	60	M	0	1	1	1	7.5
3	43	M	0	3	1	1.2	11
3	54	M	0	1	1	1	4.5
4	76	F	0	3	1	3	11.9
4	53	M	1	2	1	4	5.4
4	63	F	0	2	3	2	10.6
4	56	F	1	2	1	1	5.3
4	49	F	1	1	1	3	5

ICI—Inflammatory Cell Infiltrates; TB/CT—Trabecular Bone-to-Connective Tissue Ratio; BM—Bone Morphology; RBH—Residual Bone Height; BG—Bone Gain.

**Table 2:** Descriptive statistics of radiological parameters of patients differing in low-level laser therapy (LLLT) effect and the results of significance tests.

	LLLT		Test Results
	Yes	No	
Residual Bone Height (mm)			*U* = 39.5 *p* = 0.450 *Es* = −0.21
Mean ± SD	2.80 ± 1.25	2.31 ± 1.76	
Median [Q1, Q3]	3.0 [2.0, 4.0]	1.6 [1.0, 4.0]	
Min–Max	1.0–4.5	0.4–5.5	
Bone Gain (mm)			*U* = 50.5 *p* = 1.000 *Es* = 0.10
Mean ± SD	7.53 ± 3.32	7.02 ± 2.00	
Median [Q1, Q3]	7.3 [5.0, 10.6]	6.5 [5.6, 8.5]	
Min–Max	3.0–11.9	4.5–11.0	
Age (years)			*U* = 43.5 *p* = 0.650 *Es* = −0.13
Mean ± SD	48.6 ± 9.5	51.0 ± 11.8	
Median [Q1, Q3]	47 [44, 58]	51 [45, 55]	
Min–Max	33–60	33–76	

*U*—Mann–Whitney test statistic; *p*—significance level (*p*-value); *Es*—effect size.

**Table 3:** Descriptive statistics of radiological parameters of patients differing in graft material and the results of significance tests.

	Graft		Test Results
	Xeno	Allo	
Residual Bone Height (mm)			*U* = 61.0 *p* = 0.422 *Es* = 0.22
Mean ± SD	2.89 ± 1.76	2.00 ± 1.21	
Median [Q1, Q3]	3.0 [2.0, 4.0]	2.0 [1.1, 3.0]	
Min–Max	0.4–5.5	1.0–4.0	
Bone Gain (mm)			*U* = 44.5 *p* = 0.705 *Es* = −0.11
Mean ± SD	6.87 ± 2.67	7.68 ± 2.77	
Median [Q1, Q3]	6.0 [5.5, 9.0]	7.0 [5.3, 10.2]	
Min–Max	3.0–11.7	4.5–11.9	

*U*—Mann–Whitney test statistic; *p*—significance level (*p*-value); *Es*—effect size.

**Table 4:** Histological Assessment—Descriptive Statistics. The number (percentage) of patients in groups differing in the low-level laser therapy (LLLT) effect and histological parameters, and the results of independence tests.

	LLLT		*p*-Value
	Yes N = 10	No N = 10	
ICI:			0.029
1	6 (60%)	1 (10%)	
0	4 (40%)	9 (90%)	
TB/TC:			0.403
1	4 (40%)	7 (70%)	
2	4 (40%)	2 (20%)	
3	2 (20%)	1 (10%)	
BM:			0.356
1	8 (80%)	7 (70%)	
2	1 (10%)	3 (30%)	
3	1 (10%)	0 (0%)	
Sex:			0.035
Male	2 (20%)	7 (70%)	
Female	8 (80%)	3 (30%)	

ICI—Inflammatory Cell Infiltrates; TB/CT—Trabecular Bone-to-Connective Tissue Ratio; BM—Bone Morphology.

## Data Availability

The original data presented in the study are openly available in the open Zenodo repository under the registered no. https://doi.org/10.5281/zenodo.17807649.

## Abbreviations

The following abbreviations are used in this manuscript:

LLLT	Low-Level Laser Therapy
PBM/PBMT	Photobiomodulation/Photobiomodulation Therapy
RBH	Residual Bone Height
NBH	New Bone Height
BG	Bone Gain
ICI	Inflammatory Cell Infiltrates
TB/CT	Trabecular Bone-to-Connective Tissue Ratio
BM	Bone Morphology
BIC	Bone-to-Implant Contact
HPF	High-Power Field
